# Description of *Trichophoromyia uniniensis*, a new phlebotomine species (Diptera: Psychodidae: Phlebotominae) of Amazonas State, Brazil

**DOI:** 10.1186/1756-3305-7-400

**Published:** 2014-08-29

**Authors:** Simone Ladeia-Andrade, Nelson Ferreira Fé, Cristiani de Castilho Sanguinette, José Dilermando Andrade Filho

**Affiliations:** Laboratório de Doenças Parasitárias, Instituto Oswaldo Cruz / FIOCRUZ, Rio de Janeiro, Brazil; Fundação de Medicina Tropical Dr. Heitor Vieira Dourado (FMT-HVD), Manaus, Brazil; Grupo de Estudos em Leishmanioses, Coleção de Flebotomíneos, Centro de Referência Nacional e Internacional para Flebotomíneos, Instituto René Rachou, Fiocruz, Av. Augusto de Lima 1715, 30190-002 Belo Horizonte, MG Brasil

**Keywords:** *Trichophoromyia uniniensis* sp. nov, Sand fly, Jaú National Park, Leishmaniases

## Abstract

**Background:**

A new species of phlebotomine sand flies belonging to *Trichophoromyia* Barretto, 1962 genus is described, based on males collected in Jaú National Park, Amazonas state, Brazil.

**Methods:**

The Sand flies were mounted in Canada balsam. They were measured with a binocular Olympus CH-2 microscope with the aid of a micrometer objective and the drawings were done with the help of a *camera lucida*.

**Results:**

This new species named *Trichophoromyia uniniensis* sp. nov. is closely related to *Trichophoromyia omagua* (Martins, Llanos & Silva, 1976). The former can be distinguished from the latter by the shape of its paramere that has the lower apical region turned up in the new species.

**Conclusion:**

With the new species here described a total of 39 species belonging to the *Trichophoromyia* genus are now known, most of them present in the Amazon rainforest.

## Background

Sand flies are natural vectors of some disease agents, especially those of the leishmaniases, affecting many thousands of people worldwide [1]. The taxonomy of phlebotomines is complex due to the diversity of their morphological structures and the small differences between the species that permit precise identification. Other problems related to the taxonomy of this group are the complexes of species, morphological variations and anomalies [2–5]. Despite these difficulties, some species of sand flies have been described recently [6–13].

The genus *Trichophoromyia* is a large group of species, found mainly in rain forest [14]. Females of several species *Trichophoromyia* are morphologically similar and many species are known only by the males [15]. The medical importance of this genus is little understood, but *Trichophoromyia ubiquitalis* (Mangabeira, 1942) has been incriminated as a vector of *Leishmania* (*Viannia*) *lainsoni* Silveira, Shaw, Braga & Ishikawa, 1987 by Lainson *et al*. [16] and more recently *Trichophoromyia auraensis* (Mangabeira) has been incriminated as vector of both *L. lainsoni* and *L. braziliensis* Vianna [17].

A new species of sand flies, named *Trichophoromyia uniniensis* sp. nov., collected in Jaú National Park, Amazonas State, Brazil is described here.

## Methods

The Jaú National Park (JNP) is the largest continuous area of protected tropical rain forest in the world (2.27 million hectares). It is situated on the right bank of the Negro river, 200 km northwest of Manaus, the capital of the state of Amazonas, Northwestern Brazil (1°40’ 3°00’S, 61°26’ 64°00’W). The average annual temperature is between 26°C and 27°C, with an average annual rainfall of 2,000-2,250 mm; most of it occurring between December and April. The local population is grouped into 15 riverine communities, 9 along the Unini river (559 people, 116 dwellings) and 6 along the Jaú river (218 people, 41 dwellings).

Phlebotomine sand flies were collected during six surveys conducted between February 2009 and September 2010 in communities of JNP. CDC light traps were installed 1 meter above ground level, between 6.00 pm and 6.00 am and manual aspiration was undertaken with a Castro aspirator at the foot of trees and on other surfaces in the morning and at night.

The sand flies were mounted in Canada balsam. They were measured with a binocular Olympus CH-2 microscope with the aid of a micrometer objective and the drawings were done with the help of a *camera lucida*. The measurements are given in micrometers. The classification is that proposed by Galati [14].

In accordance with section 8.5 of the ICZN's International Code of Zoological Nomenclature, details of the new species have been submitted to ZooBank with the life science identifier (LSID) zoobank.org/References/A1273A26-8EF9-49CF-9309-35B83DD53E64.

The description of *Trichophoromyia uniniensis* nov. sp. is based on eight males. After the measurements of the holotype male, we give, in brackets, the mean, standard deviations and number of paratypes examined for each structure.

### Description

#### *Trichophoromyia uniniensis*sp. nov. Ladeia-Andrade, Fé, Sanguinette & Andrade Filho (Figures 1, 2, 3, 4and 5)

**Figure 1:**
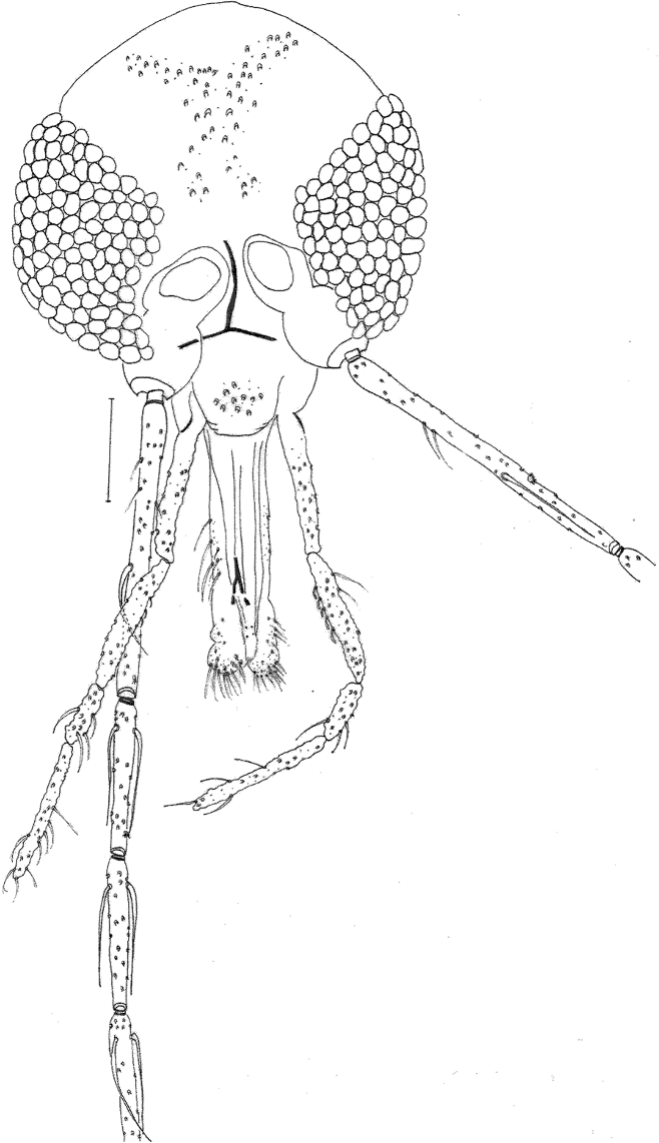
***Trichophoromyia uniniensis*** **sp. nov. (Holotype male N° 90,072).** Head, frontal view. Bar = 100 μm.

**Figure 2:**
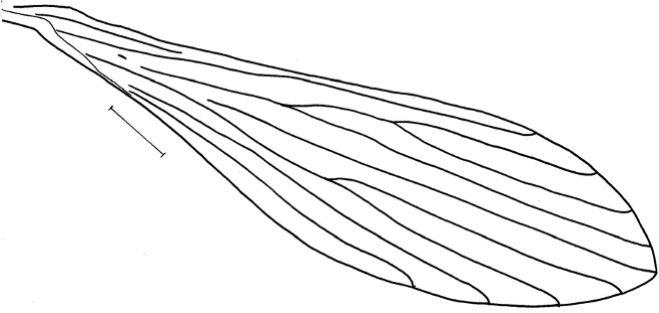
***Trichophoromyia uniniensis*** **sp. nov. (Holotype male N° 90,072).** Wing. Bar = 250 μm.

**Figure 3:**
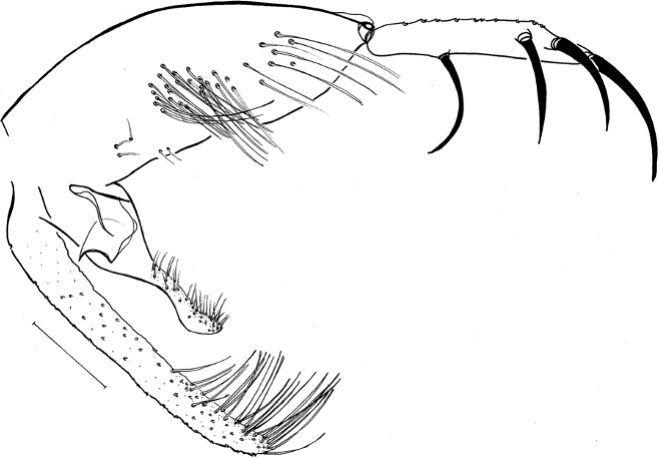
***Trichophoromyia uniniensis*** **sp. nov. (Holotype N° 90,072).** Terminalia. Bar = 100 μm.

**Figure 4:**
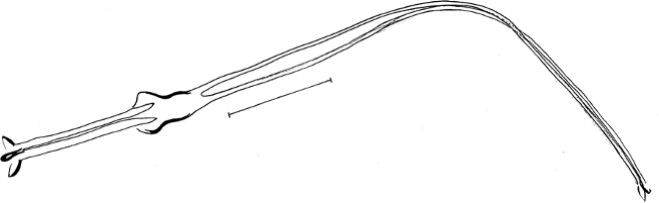
***Trichophoromyia uniniensis*** **sp. nov. (Holotype male N° 90,072).** Genital pump and filaments. Bar = 100 μm.

**Figure 5:**
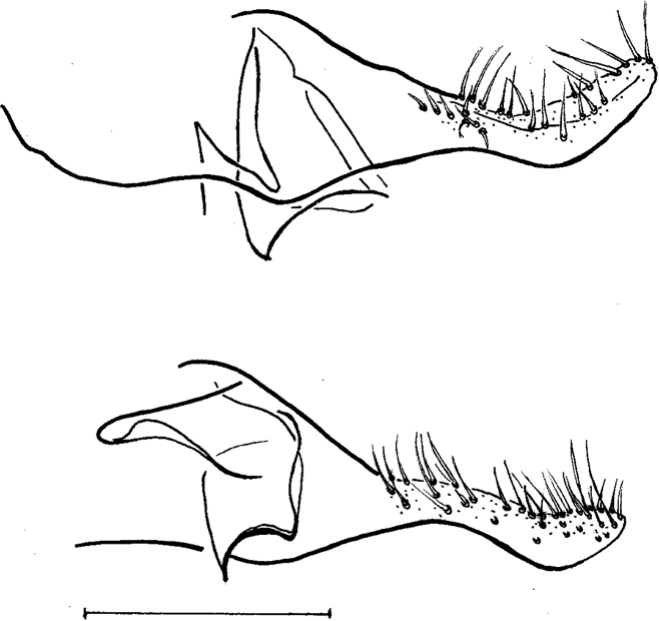
***Trichophoromyia omagua*** **(above) (from Martins et al.** [19]**);** ***Trichophoromyia uniniensis*** **(below) sp. nov. (Holotype male N° 90,072).** Paramere. Bar = 100 μm.

**Holotype (male)** Sand fly of small size, *ca.* 2,774 (2,994 ± 272.6; n = 7) in length. Scutum and paratergite light brown, contrasting with pale scutellum and pleura.

### Head (Figure 1)

552 (548 ± 6.8; n = 7) long and 345 (353 ± 11.0; n = 7) wide. Head length/head width ratio 1.60: 1 (1.53 ± 0.04; n = 7). Clypeus 82 (82 ± 3.5; n = 7) long; clypeus length/head length ratio 0.15: 1 (0.15 ± 0.01; n = 7). Eye 238 (230 ± 5.0; n = 7) long and 126 (123 ± 13.3; n = 7) wide; eye length/head length 0.43: 1 (0.42: 1 ± 0.00; n = 7). Interocular distance 112 (122 ± 7.0; n = 7). Labrum-epipharynx (LE) 201 (198 ± 3.3; n = 7). LE/head length 0.36: 1 (0.36 ± 0.01; n = 7). Antenna with simple and long ascoid, reaching the basis of the next flagellomere. Ascoids on AIII implanted at the same level. Antennal formula 2/III-XIII. Antennomere lengths: AIII 255 (260 ± 7.5; n = 6); AIV 129 (130 ± 6.4; n = 6); AV 126 (126 ± 3.1; n = 6); AXV > AXVI (AXV > AXVI; n = 4). Papilla present on AIII (pre-apical), AIV, AXIV-AXVI. Ratios: AIII/head length 0.46: 1 (0.48 ± 0.01; n = 6); AIII/LE 1.27: 1 (1.32 ± 0.04; n = 6). Palpomere lengths: P1 34 (31 ± 1.1; n = 7); P2 85 (90 ± 4.9; n = 7); P3 112 (119 ± 2.3; n = 6); P4 51 (50 ± 1.1; n = 5); and 119 (119 ± 2.4; n = 4). Palpal formula 1.4.2.3.5. [(1.4.2.3.5.; n = 1; 1.4.2.5.3.; n = 1; 1.4.2.(3.5.); n = 2]. Newstead's spines inserted medially on palpomere 3 and not visible on palpomere 2.

### Cervix

Ventrocervical sensillae absent.

### Thorax

Proepimeral setae 3–3 (3–3; n = 2; 4–4; n = 2) and anepisternal superior setae present, 18–18, in the paratypes ranging from 14 to 18, setae in the anterior region of the katepisternum absent. Wing (Figure 2) 1,863 (1,977 ± 44.6; n = 7) long and 524 (530 ± 12.5; n = 5) at maximum width. Length/width ratio 3.56: 1 (3.60: 1 ± 0.3; n = 5). Length of the vein sections: R_5_ 1,325 (1,306 ± 21.1; n = 6); *alpha* 607 (568 ± 26.9; n = 6); *beta* 235 (270 ± 15.5; n = 7); *gamma* 248 (251 ± 13.3; n = 6); *delta* 400 (352 ± 22.7; n = 6). The anterior and posterior legs of the hollotype were lost. Anterior femur, tibia, tarsomere I and tarsomeres II + III + IV + V 773 ± 11.4 (n = 4), 1,007 ± 19.3 (n = 4), 645 ± 20.5 (n = 4) and 683 ± 23.8 (n = 4), respectively. Femur, tibia, tarsomere I and tarsomeres II + III + IV + V of the median leg measurement: 718 (725 ± 7.5; n = 4), 1,270 (1,256 ± 19.3; n = 4), 745 (735 ± 30.6; n = 4) and 731 (718 ± 11.0; n = 4), respectively. The mean and standard deviation of the paratypes for femur, tibia, tarsomere I and tarsomeres II + III + IV + V of the posterior leg were 798 ± 10.1 (n = 6), 1,424 ± 10.2 (n = 6), 840 ± 16.4 (n = 6) and 791 ± 14.0 (n = 6), respectively.

### Abdomen (Figures 3, 4 and 5)

Papillae absent on abdominal tergites. Gonostyle 197 (188 ± 2.3; n = 7) long, with four spines: one apical, one upper external, one lower external and one internal implanted in the basal third. Sub terminal seta absent. Gonocoxite 330 (326 ± 4.9; n = 7) long and 116 (123 ± 8.9; n = 7) wide, about 33–35 (32–37 in paratypes) setae implanted in the middle of the structure. Paramere broad at its base, narrowing in its middle region and dilating at its apex. Distal third with ventral elbow and dorsal margin with a slight curve upwards (Figure 5). Lateral lobe 306 (300 ± 6.0; n = 7) long and 34 (29 ± 2.9; n = 7) wide, without persistent setae at its apex. Lateral lobe/gonocoxite ratio 0.93: 1 (0.90 ± 0.02; n = 7). Conical and pigmented aedeagus. Genital filament 428 (420 ± 15.2; n = 7) long and genital pump 160 (169 ± 8.0; n = 7). Genital filament/genital pump ratio 2.68: 1 (2.59 ± 0.25; n = 7). Apex of genital filaments with slight dilatation.

### Female

Unknown

### Type-material

Male holotype and all male paratypes were collected in the Jaú National Park, near the Unine river, Municipality of Barcelos, Amazonas state, Brazil with CDC light traps (Ladeia-Andrade S, Fé NF, et al. Col.). Holotype (N. 90,072) collected on 07/02/2009 in the Vila Nunes community; two males paratypes (N. 90,073 and 90,074) collected on 30/05/2009 in the Vista Alegre community; two paratypes male (N. 90,075 and 90,076) collected on 07/02/2009, in the Vila Nunes community; three paratypes male (N. 90,077, 90,078 and 90,079) collected on 06/02/2009 in the Vista Alegre community.

Holotype (90,072) and three paratypes (N. 90,073, 90,075 and 90,077) deposited in the "Coleção de Flebotomíneos" of the "Instituto René Rachou/FIOCRUZ" (FIOCRUZ-COLFLEB), Belo Horizonte, Brazil. Two paratypes (N. 90,074 and 90,078) deposited in the collection of "Instituto Nacional de Pesquisas da Amazônia (INPA), Manaus, Brazil. Two paratypes (90,076 and 90,079) deposited in the collection of "Instituto Oswaldo Cruz/FIOCRUZ, Rio de Janeiro, Brazil.

### Etymology

The name *Trichophoromyia uniniensis* sp. nov. alludes to the Unini river, located in the Jaú National Park, the type locality of this species.

## Results and discussion

To date, 38 species have been formally described for the genus *Trichophoromyia* [13, 14, 18] which can be divided into two groups, based on the ratio between the genital filaments/genital pump. Some species have this ratio lower than 3 and others greater than 4. The new species, with a ratio of 2.68: 1 (2.59 ± 0.25; n = 7) is included in the first group. In *Trichophoromyia* genus, only four species have this characteristic: *Trichophoromyia meirai* (Causey & Damasceno, 1945), *Trichophoromyia omagua* (Martins, Llanos & Silva, [19]), *Trichophoromyia reburra* (Fairchild & Hertig, 1961) and *T. ubiquitalis. Trichophoromyia meirai* present the lateral lobe longer than the gonocoxite, while in the new species the opposite occurs. The paramere can be used to distinguish *T. ubiquitalis* and *T. reburra* from *T. uniniensis*. This structure is slightly curved towards the gonocoxite in the new species and straight in the others species. *Trichophoromyia omagua* also presents the curved paramere [19]. However, this species can be distinguished from the *T. uniniensis* n. sp. by the shape of the paramere (see Figure 5). The dorsal margin of the paramere in *T. omagua* is concave while in the new species in its median it is slightly convex, followed by a small pre-apical concavity. Further, the elbow in the ventral region of *T. omagua* is closer to the middle of the paramere while in the new species it is situated near its fourth apical region.

Only 22 species of *Trichophoromyia* are described, based on both sexes. This because females of several species present similar morphology. [15]. In fact, of the 22 species whose females have been described, only those of *T. reburra*, *Trichophoromyia cellulana* (Young & Duncan, 1979), *T. omagua* and *T. ubiquitalis* can be differentiated morphologically [14]. Some females of *Trichophoromyia* were collected in the Jaú National Park, however, we prefer not to describe them as females of *Trichophoromyia uniniensis* since other species of *Trichophoromyia* were also collected in sympatry with the new species.

## Conclusion

With the new species here described a total of 39 species belonging to the *Trichophoromyia* genus are now known, most of them present in the Amazon rainforest.
